# Chemical Characterization of *Marrubium vulgare* Volatiles from Serbia

**DOI:** 10.3390/plants10030600

**Published:** 2021-03-23

**Authors:** Milica Aćimović, Stefan Ivanović, Katarina Simić, Lato Pezo, Tijana Zeremski, Jelena Ovuka, Vladimir Sikora

**Affiliations:** 1 Institute of Field and Vegetable Crops Novi Sad, Maksima Gorkog 30, 21000 Novi Sad, Serbia; tijana.zeremski@ifvcns.ns.ac.rs (T.Z.); jelena.ovuka@ifvcns.ns.ac.rs (J.O.); vladimir.sikora@ifvcns.ns.ac.rs (V.S.); 2 Institute of Chemistry, Technology and Metallurgy, University of Belgrade, 11000 Belgrade, Serbia; stefan.ivanovic@ihtm.bg.ac.rs (S.I.); katarina.simic@ihtm.bg.ac.rs (K.S.); 3 Institute of General and Physical Chemistry, University of Belgrade, 11000 Belgrade, Serbia; latopezo@yahoo.co.uk

**Keywords:** horehound, GC–MS, retention indices, QSRR, boosted trees regression model

## Abstract

*Marrubium vulgare* is a cosmopolitan medicinal plant from the Lamiaceae family, which produces structurally highly diverse groups of secondary metabolites. A total of 160 compounds were determined in the volatiles from Serbia during two investigated years (2019 and 2020). The main components were *E*-caryophyllene, followed by germacrene D, *α*-humulene and *α*-copaene. All these compounds are from sesquiterpene hydrocarbons class which was dominant in both investigated years. This variation in volatiles composition could be a consequence of weather conditions, as in the case of other aromatic plants. According to the unrooted cluster tree with 37 samples of *Marrubium* sp. volatiles from literature and average values from this study, it could be said that there are several chemotypes: *E*-caryophyllene, *β*-bisabolene, α-pinene, β-farnesene, *E*-caryophyllene + caryophyllene oxide chemotype, and diverse (unclassified) chemotypes. However, occurring polymorphism could be consequence of adaptation to grow in different environment, especially ecological conditions such as humidity, temperature and altitude, as well as hybridization strongly affected the chemotypes. In addition, this paper aimed to obtain validated models for prediction of retention indices (RIs) of compounds isolated from *M. vulgare* volatiles. A total of 160 experimentally obtained RIs of volatile compounds was used to build the prediction models. The coefficients of determination were 0.956 and 0.964, demonstrating that these models could be used for predicting RIs, due to low prediction error and high *r*^2^.

## 1. Introduction

*Marrubium vulgare* L., also known as white horehound, is a perennial species from the Lamiaceae family. It is indigenous to the region between the Mediterranean Sea and Central Asia; however, today it is found worldwide, apart from the coldest regions and high altitudes [1]. This plant is highly resistant to drought and due to this it grows well in semiarid areas [2]. Additionally, as it is a moderate salt-tolerant species this medicinal plant could be grown on saline soil [3]. The surface of *M. vulgare* vegetative and generative organs is densely clothed with glandular and nonglandular trichomes which accumulate secondary metabolites [4]. *M. vulgare* produces structurally highly diverse groups of secondary metabolites, thus represents a valuable source of bioactive compounds and preparations with health-promoting effects: antioxidant, hepatoprotective, antiproliferative, anti-inflammatory, antidiabetic, and antimicrobial [5]. The use of this herb in traditional medicine is recorded worldwide for ameliorating chronic cough and cold, numerous conditions related to skin, liver, gastric, heart, and immune system [6]. Generally, *M. vulgare* is poor in essential oil, and the major compounds are diverse [1,7,8,9,10,11,12,13,14,15,16,17]. This proves that there are various chemotypes of *M. vulgare*. The lack of information in this field is pointed out by Yabrir [1,18]. The studies about genus *Marrubium* are mainly focused on taxonomical, morphological, and genetic diversity [4,19,20,21,22,23,24,25,26].

The main aim of this investigation was to determine volatiles composition of *M. vulgare* grown in Serbia during two years and to compare its chemical composition with literature data not only of *M. vulgare* but with other species from this genus as well (*M. anisodon*, *M. aschersonii*, *M. astracanicum*, *M. crassidens*, *M. deserti*, *M. duabense*, *M. parviflorum*, *M. peregrinum*, *M. persicum*, *M. propinquum*, *M. velutinum*). Another goal was to establish the new quantitative structure retention relationship (QSRR) models for anticipating the retention indices (RIs) of certain compounds in *M. vulgare* volatiles obtained by GC–MS chromatography utilizing the genetic algorithm (GA) variable selection method and the boosted trees regression. Furthermore, we gather information about the volatile compounds of species from *Marrubium* genus in order to classify the chemotype of *M. vulgare* from this study according to unrooted cluster tree.

## 2. Results

The main components in *M. vulgare* volatiles were *E*-caryophyllene with 24.6% and 23.0%, followed by germacrene D with 9.6% and 17.0%, *α*-humulene with 5.2% and 5.3% as well as *α*-copaene with 3.3% and 6.1% in 2019 and 2020, respectively. All these compounds are from the sesquiterpene hydrocarbons class which was dominant in both years of the investigation, 52.0% in 2019 and 67.8% in 2020. This variation in volatiles composition could be a consequence of weather conditions, as in case of other aromatic plants [27,28,29,30,31,32,33].

However, some of the components detected in *M. vulgare* volatiles during the two-year research have not been detected yet in this species, while other components have not been detected in other species of this genus. ScienceDirect Elsevier, SpringerLink, PubMed, Scopus, Scifnder, Web of Science, Wiley Online, and Google Scholar databases were reviewed and scientific publications from 1990 until 2020 that deal with chemical composition of volatiles species from genus *Marrubium* were summarized and shown in Table 1.

**Figure 1:**
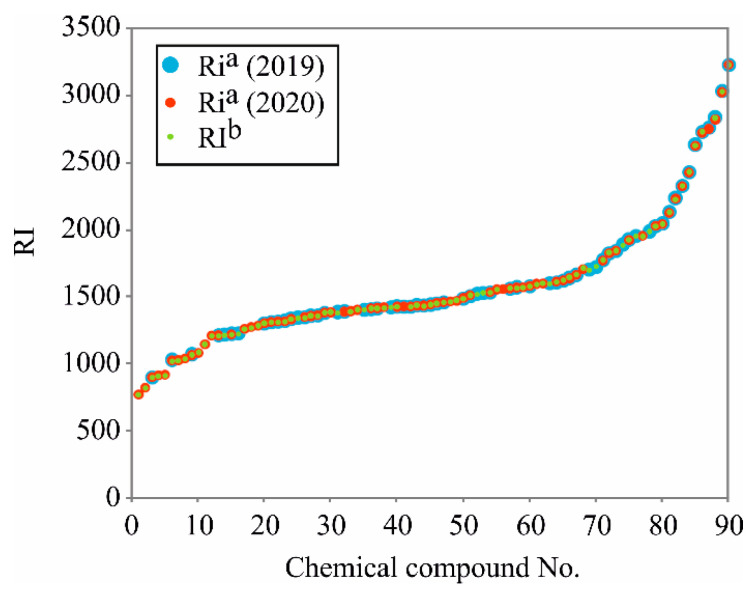
Retention indices (RIs) of the *M. vulgare* volatiles composition, from experimentally obtained GC–MS data on a HP-5MS column (RI^a^) and NIST database (RI^b^).

The predicted RIs are shown in Table 1, and confirm the good quality of the constructed BRT model by showing the relationship between the predicted and experimental RI values. Graphical comparison between experimentally obtained RIs of *M. vulgare* volatiles composition (RI^a^), the retention time indices found in NIST database (RI^b^) and the retention time indices predicted by the two BRT models (RI_pred._) are presented in Figure 1. 

In order to calculate the molecular descriptors, the PaDel-descriptor was used in this investigation. Due to a great amount of data that was obtained, it was required to select the most important set of descriptors to build the adequate model which would be able to predict the RIs [55]. The factor analysis was done before the GA calculation, and only ca. 320 uncorrelated descriptors remained in the GA calculation [56,57]. The seven most significant molecular descriptors chosen by GA are as follows: four 2D autocorrelation descriptors (AATSC4e, AATSC2p, GATS6v and MATS5v), two Barysz matrix descriptors (VR1_Dzs and SM1_Dzv) and Vertex adjacency information (magnitude) descriptor (VAdjMat).

**Figure 2:**
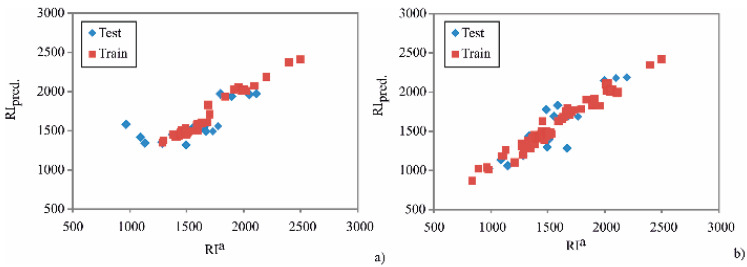
Comparison of experimentally obtained RIs on a HP-5MS column (RIa) with BRT pre-dicted values (RIpred.) in 2019 (**a**) and 2020 (**b**).

The predicted RIs and molecular descriptors are presented in Table 1. Seven molecular descriptors were utilized for predictions of RIs in the two BRT models. The predicted RIs are presented in Figure 2, and visually confirm the adequate prediction capabilities of the constructed BRT by showing the relationship between the predicted and experimental retention values.

Separation of compounds in GC–MS and their RIs is linked to their affinity towards mobile and stationary phases. Affinity and solubility of separated molecules directly depend on their chemical structure and physico-chemical properties, which could be expressed by molecular descriptors. According to Pearson’s correlation coefficients, there was a rather poor correlation between all 3D autocorrelation descriptors (Table 2). Therefore, utilized molecular descriptors were appropriate to predict RIs of compounds in *M. vulgare* by the two multivariate BRT models [58].

Detailed explanations about the descriptors were found in the Handbook of Molecular Descriptors [59]. These descriptors encode different aspects of the molecular structure and were applied to develop the QSRR model. According to Pearson’s correlation, there was a rather poor correlation between all molecular descriptors. Hence, utilized descriptors were appropriate to predict RIs of compounds isolated from *M. vulgare* volatiles by the two multivariate BRT models. The calibration and predictive capability of a QSRR model should be tested through model validation. The most widely used squared correlation coefficient (*r*^2^) can provide a reliable indication of the fit of the model, thus, it was employed to validate the calibration capability of a QSRR model.

In order to explore the nonlinear relationship between RIs and the descriptors selected by GA, BRT technique was used to build the two predictive models. Two BRT models were constructed to predict the retention time of compounds isolated from *M. vulgare* volatiles, respectively. The coefficients of determination were 0.956 and 0.964, respectively, indicating that these models could be used for prediction of RIs, due to low prediction error and high *r*^2^. The tests of the two BRT models fit (2019 and 2020) are shown in Table 3, with the higher *r*^2^ values and lower χ^2^, MBE, RMSE, and MPE values showing the better fit to the experimental results [60,61].

**Figure 3:**
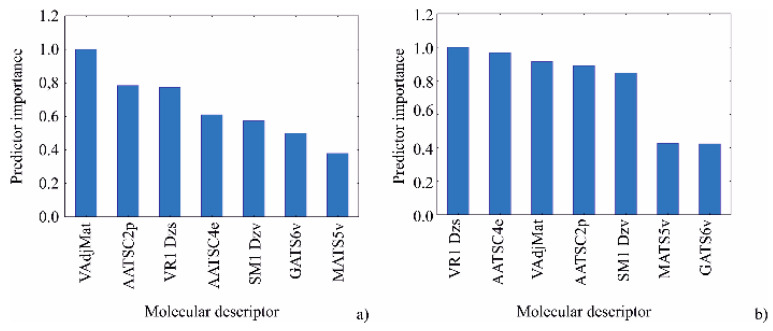
Predictor importance of the molecular descriptors on RI in 2019 (**a**) and 2020 (**b**).

Obtained results reveal the reliability of the BRT models for predicting the RIs of compounds in *M. vulgare* volatiles obtained by GC–MS analysis. The influence of the seven most important molecular descriptors, identified by using genetic algorithm on the RIs was studied in this section. According to the Figure 3, VAdjMat was the most important molecular descriptor for chemical compounds’ RIs calculation in *M. vulgare*, during 2019, while VR1 Dzs was the most important variable during 2020.

## 3. Discussion

**Figure 4:**
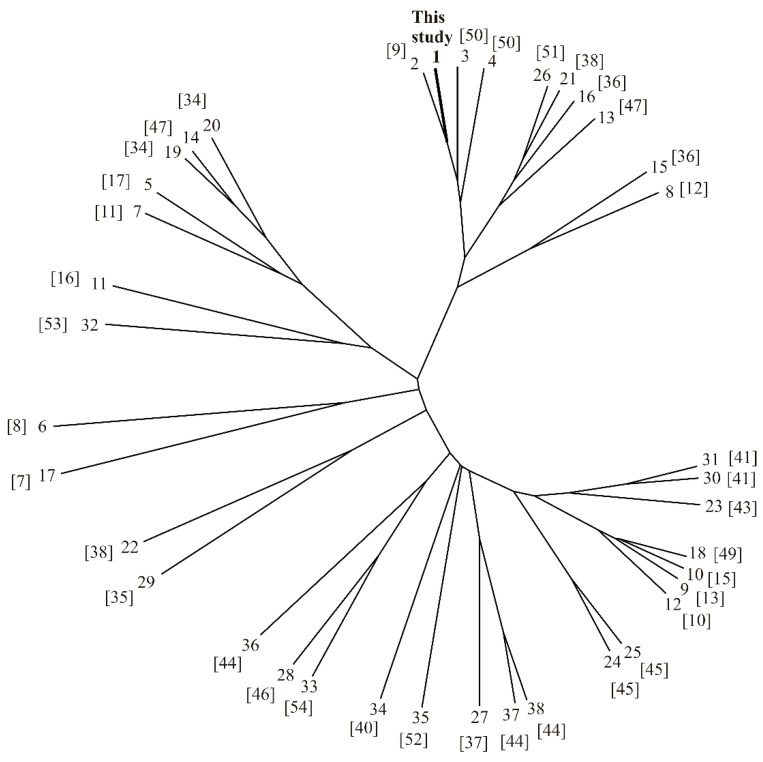
Unrooted cluster tree for different *Marrubium* samples.

According to the cluster analysis (unrooted cluster tree) with 37 samples of *Marrubium* sp. volatiles from literature and average values from this study (Figure 4), it could be said that there are several chemotypes, but only *E*-caryophyllene chemotype [9,12,36,38,47,50,51] is clearly segregated. However, these are samples of *M. vulgare*, *M. incanum*, *M. parviflorum*, *M. peregrinum,* and *M. crassidens* grown in Serbia, Poland, Slovakia, Egypt, and Iran. This indicated that genus *Marrubium*is very diverse in the case of volatiles composition. 

In addition, *E*-caryophyllene is a compound which is occurring in all samples (except *M. vulgare* from Eastern Algeria [15]), but in *E*-caryophyllene chemotype its content ranges between 15.6% and 45.8%. Other chemotypes can be classified as *β*-bisabolene (13.1–28.3%) [11,17,34,47], α-pinene (21.5–28.9%) [16,53], β-farnesene (20.2–24.2%) [37,44], *E*-caryophyllene + caryophyllene oxide chemotype [44,46,54], and diverse (unclassified) chemotypes [7,8,10,13,15,35,38,40,41,43,45,49,52].

Occurring polymorphism could be a consequence of adaptation to grow in different environments [19,62], especially ecological conditions such as humidity, temperature and altitude [22] as well as hybridization [20] strongly affected the chemotypes, as well as biotic and abiotic stresses (including temperature, light, water, salt, and oxidative stresses) [63].

Detected compounds in *M. vulgare* volatiles obtained by GC–MS analysis were used for QSRR analysis. The following seven molecular descriptors that characterize the RIs of obtained compounds were suggested by the genetic algorithm. Selected molecular descriptors were not autocorrelated which was suggested by a correlation coefficient matrix; thus, descriptors were suitable for QSRR analysis. These descriptors were utilized as inputs for the boosted trees regression models, for estimating the RIs using a set of GC–MS data from a series of 160 compounds found in *M. vulgare* volatiles. Statistical models that quantify the relation between the structure of molecules and their chromatographic RIs were represented by the quantitative structure retention relationship (QSRR) model [58,64]. Numerous publications related to the QSRR analysis in plants from Lamiaceae family could be found in the literature: *Thymus vulgaris* [65], *T. serpyllum* [66], *Satureja kitaibelii* [55], *Salvia officinalis* [67], as well as *Stachys* sp. [68]. The connection between the molecular descriptors and the retention time can be established by artificial neural network, machine learning algorithms [69,70,71,72,73] or by boosted trees regression (BRT) [74].

## 4. Materials and Methods

### 4.1. Plant Material

*M. vulgare* was grown in the Institute of Field and Vegetable Crops (IFVCNS) collection garden of medicinal and aromatic plants in Bački Petrovac (45°21′ N; 19°35′ E), confirmed by Milica Rat, PhD, and deposited at the Herbarium BUNS (Department of Biology and Ecology, Faculty of Sciences, University of Novi Sad) under the voucher number 2-1409. After seed maturation (August 2018), it was collected and sown in field conditions in September 2018 and 2019. The experimental plot was 10 m long and 5 m wide, with a 70 cm spacing between rows. From the seven rows, only three central rows were used for collecting plant material to avoid edge effects (one row one sample).

### 4.2. Volatiles Isolation and Analysis

Flowering aerial parts of *M. vulgare* (*Marrubii herba*) were collected during July 2019 and 2020, dried in a solar dryer at a temperature of 40° with air circulation. After drying, plant material was fragmented, and volatiles was extracted by Clevenger apparatus. Taking in account that *M. vulgare* produces trace amounts of essential oil, it was trapped in n-hexane. This process was performed in tree repetition for both years, as well as analysis of chemical composition.

GC–MS analysis was carried out using an Agilent 7890A apparatus equipped with a 5975 C MSD, FID and a nonpolar HP-5MS fused-silica capillary column (30 m × 0.25 mm, film thickness 0.25 μm). The carrier gas was helium, and its inlet pressure was 19.6 psi and linear velocity of 1 mL/min at 210 °C. The injector temperature was 250 °C, injection volume was 1 μL, split ratio, 10:1. Mass detection was carried out under source temperature conditions of 230 °C and interface temperature of 315 °C. The EI mode was set at electron energy, 70 eV with mass scan range of 40–600 amu. Temperature was programmed from 60 to 300 °C at a rate of 3 °C/min. The components were identified based on their linear retention index relative to C8-C32 n-alkanes, compared with data reported in the literature (Adams4 and NIST11 databases). The relative percentage of the oil constituents was expressed as percentages by FID peak area normalization.

### 4.3. QSRR Analysis

PaDel-descriptor software was used to calculate specified molecular descriptors [75], as described in our previous investigation [66]. Factor analysis and genetic algorithm (GA) were used to determine the most important descriptors [76,77]. The relationship between the chosen descriptors was examined and collinear descriptors were excluded. Statistica 10 software was used for the statistical investigation of the data [78].

### 4.4. BRT Model

In order to relate and to predict categorical or continuous dependent variables the BRT model could be used [79,80], as it does not require transformation or outliers [81]. The BRT method calculation is connected to the boosting methods enforced to regression trees [82]. The main idea is to calculate a set of simple trees, where each successive tree is built for the prediction residuals of the preceding tree [83]. This method builds binary trees such as partition the data into two samples at each split node [78].

The decision trees are combined through a cross-validation or “boosting” procedure in order to acquire the single computational model [84]. BRT modeling consists of the following steps: (a) an initial regression tree is defined according to a minimum loss function; (b) the other trees are engaged in the iterative process in which several new regression trees were developed and selected to the subsequent according to the StatSoft Statistica’s recommendation—the least square error (LSE); (c) step (b) is repeated until a stopping criterion is reached (for instance, the value of LSE). 

In this study, several regularization parameters were set in order to optimize the fit between experimental results and computing model: the number of trees (between 100 and 1000), learning rate (between 0.0005 and 0.1), random test data proportion (0.1–0.9) and subsample proportion (0.1–0.9). According to Statistica’s manual, prior to computation, a subsample of data is created, according to random test data proportion of the cases, and these data are treated as test samples used to evaluate the appropriate fit of the model. The remaining set of data is used for the analyses via stochastic gradient boosting (for the selection of samples for consecutive boosting steps).

### 4.5. Cluster Analysis

The cluster analysis (CA) was used to evaluate intra- and interpopulation variability and differentiation of volatile constituents of *Marrubium* samples collected in different locations and/or taken from literature reports. The phylogenetic tree diagram for *Marrubium* samples was calculated and plotted using R software 4.0.3 (64-bit version). The R package “ape” (Analysis of Phylogentics and Evolution) was used for calculation, applied as a graphical tool to represent the arrangements of similar volatiles concentration (evaluated in the cluster analysis). The obtained experimental results were collected in the matrix, after which the hierarchical cluster analysis was performed. The distance matrix was determined using the Euclidean method, while the cluster analysis was performed using the “complete” method.

## 5. Conclusions

The main components in *M. vulgare* volatiles were *E*-caryophyllene (24.6% and 23.0%), followed by germacrene D (9.6% and 17.0%), *α*-humulene (5.2% and 5.3%) and *α*-copaene (3.3% and 6.1%) in 2019 and 2020, respectively. All these compounds are from sesquiterpene hydrocarbons class, which was dominant in both years of the investigation, 52.0% in 2019 and 67.8% in 2020.

The results demonstrated that the boosted trees regression models were adequate in predicting the RIs of the compounds in *M. vulgare* volatiles obtained by GC–MS analysis on a HP-5MS column. The coefficients of determination were 0.956 and 0.964 (for compounds found in *M. vulgare* volatiles, during the years 2019 and 2020, respectively), which is a good indication that these models could be used as a fast mathematical tool for prediction of RIs, due to low prediction error and moderately high *r*^2^. Suitable models with high statistical quality and low prediction errors were derived, and it could be further used for estimation of RIs of newly detected compounds.

According to the unrooted cluster tree with 37 samples of *Marrubium* sp. volatiles from literature and average values from this study, it could be said that there are several chemotypes: *E*-caryophyllene, *β*-bisabolene, α-pinene, β-farnesene, *E*-caryophyllene + caryophyllene oxide chemotype, and diverse (unclassified) chemotypes. However, occurring polymorphism could be a consequence of adaptation to grow in different environments, especially ecological conditions such as humidity, temperature and altitude, as well as hybridization which strongly affected the chemotypes. Further research on *M. vulgare* chemotypes needs to be focused on genetic markers, because evaluation of genetic diversity has key importance in improving the quality of raw material used for industrial purposes.

## Figures and Tables

**Table 1:** Chemical composition of *Marrubium vulgare* during two years (2019 and 2020).

No	Compound/Class	Cycle	RI_pred._	2019		2020		Reference
				RI^a^	%	RI^a^	%	
1	2E-Hexenal O	Train	892.915	-	-	847	0.2	*M. aschersonii* [34], *M. deserti* [35], *M. peregrinum* [36], *M. vulgare* [10,12,15,16,34]
2	Furan, 2,5-diethyltetrahydro O	Validation	853.684	-	-	897	0.1	
3	1-Octen-3-ol O	Validation	965.818	976	0.2	974	0.6	*M. anisodon* [37], *M. astracanicum* [38], *M. crassidens* [39], *M. deserti* [35], *M. duabense* [40], *M. parviflorum* [41,42], *M. peregrinum* [43,44], *M. persicum* [45], *M. propinquum* [41], *M. velutinum* [44], *M. vulgare* [7,8,10,15]
4	2-Pentyl furan O	Train	1059.803	-	-	989	0.1	
5	3-Octanol O	Test	962.233	-	-	992	0.1	*M. anisodon* [37], *M. astracanicum* [46], *M. duabense* [40], *M. peregrinum* [36,44], *M. velutinum* [44]
6	Linalool OMN	Train	1106.041	1102	0.1	1098	0.1	*M. aschersonii* [34], *M. astracanicum* [46], *M. parviflorum* [41,42,47,48], *M. peregrinum* [36,43,44], *M. persicum* [45], *M. velutinum* [44], *M. vulgare* [8,10,12,17,34,36,47,49]
7	n-Nonanal O	Train	1078.484	-	-	1102	0.1	*M. aschersonii* [34], *M. deserti* [35], *M. duabense* [40], *M. peregrinum* [43,44], *M. persicum* [45], *M. velutinum* [44], *M. vulgare* [34]
8	E-Thujone OMN	Train	1118.307	-	-	1114	0.1	*M. peregrinum* [43], *M. vulgare* [8,15]
9	NI-1	-	-	-	-	1132	0.1	-
10	Geijerene O	Train	1192.301	1143	0.1	1139	0.6	*M. incanum* [50,51], *M. parviflorum* [42,47], *M. peregrinum* [43], *M. vulgare* [50]
11	2E-Nonen-1-al O	Validation	1097.602	-	-	1156	0.1	
12	β-Cyclocitral O	Train	1216.889	-	-	1219	0.1	*M. peregrinum* [44], *M. velutinum* [44], *M. vulgare* [10]
13	Cogeijerene O	Train	1203.235	-	-	1283	0.1	
14	Pregeijerene O	Train	1149.857	1290	0.1	1287	0.2	*M. astracanicum* [38], *M. crassidens* [38], *M. parviflorum* [42,47], *M. peregrinum* [43]
15	Thymol AR	Test	1209.017	1292	0.3	-	-	*M. deserti* [52], *M. vulgare* [7,8,10,15,50]
16	2-Undecanone O	Train	1269.228	1295	0.1	1292	Trace	*M. vulgare* [15]
17	Carvacrol AR	Validation	1179.072	1302	0.1	-	-	*M. duabense* [40], *M. incanum* [50], *M. parviflorum* [42], *M. peregrinum* [43], *M. vulgare* [7,8,10,49,50]
18	δ-Elemene ST	Test	1512.436	-	-	1336	0.1	*M. anisodon* [37], *M. astracanicum* [38], *M. crassidens* [38], *M. deserti* [35,40], *M. duabense* [40], *M. incanum* [50,51], *M. parviflorum* [47], *M. peregrinum* [44], *M. persicum* [53], *M. thessalum* [54], *M. velutinum* [44], *M. vulgare* [47,50]
19	α-Cubebene ST	Train	1491.202	-	-	1348	0.1	*M. astracanicum* [38], *M. crassidens* [39], *M. deserti* [35,40], *M. duabense* [40], *M. parviflorum* [47], *M. peregrinum* [44], *M. persicum* [45], *M. vulgare* [8,47]
20	Eugenol AR	Train	1372.624	-	-	1357	0.4	*M. aschersonii* [34], *M. peregrinum* [36,43,44], *M. persicum* [53], *M. velutinum* [44], *M. vulgare* [10,12,34,36,47]
21	α-Copaene ST	Train	1475.878	1377	3.3	1377	6.1	*M. anisodon* [37], *M. aschersonii* [34], *M. astracanicum* [38], *M. crassidens* [38], *M. deserti* [35], *M. duabense* [40], *M. incanum* [50,51], *M. parviflorum* [42,47,48], *M. peregrinum* [36,43,44], *M. persicum* [53], *M. thessalum* [54], *M. velutinum* [44], *M. vulgare* [8,9,10,11,12,13,36,47,50]
22	β-Bourbonene ST	Train	1487.976	1385	0.8	1384	1.2	*M. anisodon* [37], *M. astracanicum* [38], *M. crassidens* [38], *M. deserti* [35,52], *M. incanum* [50,51], *M. parviflorum* [41,42,47,48], *M. peregrinum* [43,44], *M. persicum* [45,53], *M. thessalum* [54], *M. velutinum* [44], *M. vulgare* [9,10,13,50]
23	NI-2	-	-	-	-	1388	0.1	-
24	β-Cubebene ST	Test	1475.610	1390	0.1	1389	0.2	*M. aschersonii* [34], *M. deserti* [35], *M. peregrinum* [43,44], *M. parviflorum* [42], *M. velutinum* [44], *M. vulgare* [12,13,34,47]
25	β-Elemene ST	Train	1475.506	1392	0.4	1391	1.0	*M. anisodon* [37], *M. astracanicum* [38], *M. crassidens* [38], *M. deserti* [35,52], *M. duabense* [40], *M. incanum* [50,51], *M. parviflorum* [42,47], *M. peregrinum* [44], *M. persicum* [53], *M. thessalum* [54], *M. velutinum* [44], *M. vulgare* [47,50]
26	Z-Caryophyllene ST	Train	1463.161	1407	0.1	1406	0.2	
27	α-Z-Bergamotene ST	Train	1428.215	1416	0.2	-	-	
28	E-Caryophyllene ST	Validation	1463.161	1422	24.6	1423	23.0	*M. anisodon* [37], *M. aschersonii* [34], *M. astracanicum* [38,46], *M. crassidens* [38,39], *M. deserti* [35,52], *M. duabense* [40], *M. incanum* [50,51], *M. parviflorum* [41,42,47,48], *M. peregrinum* [36,43,44], *M. persicum* [45,53], *M. propinquum* [41], *M. thessalum* [54], *M. velutinum* [44], *M. vulgare* [7,8,9,10,11,12,13,16,17,34,36,47,49,50]
29	β-Copaene ST	Test	1459.623	1430	0.4	1430	1.3	*M. incanum* [50], *M. vulgare* [50]
30	α-E-Bergamotene ST	Train	1428.215	1436	0.1	1435	0.1	*M. anisodon* [37], *M. astracanicum* [46], *M. crassidens* [38], *M. parviflorum* [42,47], *M. peregrinum* [44], *M. velutinum* [44], *M. vulgare* [47]
31	NI-3	-	-	1445	0.2	1444	0.6	-
32	α-Humulene ST	Validation	1503.999	1454	5.2	1455	5.3	*M. anisodon* [37], *M. aschersonii* [34], *M. astracanicum* [38,46], *M. crassidens* [38,39], *M. duabense* [40], *M. incanum* [50,51], *M. parviflorum* [42,47], *M. peregrinum* [36,43], *M. persicum* [45], *M. thessalum* [54], *M. velutinum* [44], *M. vulgare* [8,9,10,12,13,15,34,36,47,50]
33	Sesquisabinene ST	Train	1442.740	-	-	1457	0.9	
34	E-β-Farnesene ST	Test	1431.419	1457	1.3	-	-	*M. anisodon* [37], *M. aschersonii* [34], *M. crassidens* [39], *M. parviflorum* [41,42,47], *M. peregrinum* [43,44], *M. persicum* [45], *M. propinquum* [41], *M. thessalum* [54], *M. velutinum* [44], *M. vulgare* [8,10,12,16,17,34,36,47]
35	C16H34 A	Train	1573.436	1462	1.5	1462	0.2	
36	NI-4	-	-	-	Trace	-	0.2	-
37	Z-Muurola-4(14),5-diene ST	Train	1482.433	-	-	1466	0.1	
38	NI-5	-	-	1469	0.1	-	-	-
39	NI-6	-	-	1472	0.1	-	-	-
40	E-Cadina-1(6),4-diene ST	Train	1481.465	-	-	1475	Trace	*M. vulgare* [15]
41	γ-Muurolene ST	Test	1450.203	1479	0.1	-	-	*M. incanum* [50], *M. peregrinum* [43,44], *M. parviflorum* [42], *M. velutinum* [44]
42	Germacrene D ST	Test	1450.188	1483	9.6	1487	17.0	*M. anisodon* [37], *M. aschersonii* [34], *M. astracanicum* [38], *M. crassidens* [38,39], *M. deserti* [35,52], *M. incanum* [50,51], *M. parviflorum* [41,42,47,48], *M. peregrinum* [36,43,44], *M. persicum* [45,53], *M. propinquum* [41], *M. thessalum* [54], *M. velutinum* [44], *M. vulgare* [9,10,11,12,13,15,16,17,34,36,47,50]
43	E-β-Ionone O	Test	1471.735	1486	0.4	1489	Trace	*M. anisodon* [37], *M. aschersonii* [34], *M. duabense* [40], *M. incanum* [51], *M. parviflorum* [42], *M. peregrinum* [43,44], *M. thessalum* [54], *M. vulgare* [12]
44	NI-7	-	-	-	-	1489	0.1	-
45	epi-Cubebol OST	Train	1622.285	-	-	1495	0.2	
46	Viridiflorene ST	Validation	1507.447	1497	0.1	-	-	
47	Bicyclogermacrene ST	Validation	1493.697	1498	0.2	1498	0.2	*M. astracanicum* [38], *M. crassidens* [38,39], *M. deserti* [35,52], *M. duabense* [40], *M. incanum* [50,51], *M. parviflorum* [41,42,47,48], *M. peregrinum* [36,43,44], *M. persicum* [45], *M. propinquum* [41], *M. thessalum* [54], *M. velutinum* [44], *M. vulgare* [10,11,17,50]
48	NI-8	-	-	-	-	1499	0.7	-
49	Pentadecane A	Test	1486.884	1500	0.2	1500	Trace	
50	α-Muurolene ST	Train	1465.650	1501	0.1	1501	0.2	*M. aschersonii* [34], *M. deserti* [35,52], *M. incanum* [51], *M. peregrinum* [43], *M. velutinum* [44], *M. vulgare* [12,13,34]
51	Germacrene A ST	Train	1450.188	1508	0.1	1506	0.1	*M. incanum* [50], *M. parviflorum* [47,48]
52	β-Bisabolene ST	Validation	1425.139	1511	0.2	1507	0.2	*M. anisodon* [37], *M. aschersonii* [34], *M. crassidens* [38], *M. parviflorum* [47], *M. peregrinum* [44], *M. persicum* [45], *M. propinquum* [41], *M. thessalum* [54], *M. velutinum* [44], *M. vulgare* [11,12,13,17,34,47,49]
53	γ-Cadinene ST	Test	1450.203	1513	0.2	1515	0.4	*M. deserti* [52], *M. incanum* [50], *M. parviflorum* [47,48], *M. peregrinum* [43,44], *M. persicum* [53], *M. velutinum* [44], *M. vulgare* [7,10,15,47]
54	δ-Cadinene ST	Test	1475.070	1523	4.7	1528	9.7	*M. deserti* [52], *M. incanum* [50], *M. parviflorum* [42,47], *M. peregrinum* [43,44], *M. persicum* [53], *M. velutinum* [44], *M. vulgare* [7,10,15,47]
55	E-Cadina-1,4-diene ST	Train	1471.521	1533	0.1	1533	0.1	*M. vulgare* [15]
56	α-Cadinene ST	Train	1465.650	-	-	1537	0.1	*M. peregrinum* [43,44], *M. velutinum* [44], *M. vulgare* [47]
57	α-Calacorene ST	Train	1540.123	-	-	1543	0.1	*M. deserti* [52], *M. vulgare* [12,15]
58	NI-9	-	-	1555	0.2	1552	0.2	-
59	E-Nerolidol OST	Validation	1567.136	1561	3.5	1564	1.5	*M. anisodon* [37], *M. deserti* [52], *M. parviflorum* [42], *M. peregrinum* [43,44], *M. thessalum* [54], *M. velutinum* [44], *M. vulgare* [9,36]
60	NI-10	-	-	-	-	1571	0.1	-
61	NI-11	-	-	1577	0.2	1575	0.9	-
62	NI-12	-	-	-	-	1582	0.3	-
63	Caryophyllene oxide OST	Test	1636.612	1580	1.0	1583	1.8	*M. anisodon* [37], *M. astracanicum* [46], *M. crassidens* [38,39], *M. deserti* [52], *M. duabense* [40], *M. incanum* [50,51], *M. parviflorum* [41,42,47,48], *M. peregrinum* [36,43], *M. persicum* [45,53], *M. propinquum* [41], *M. thessalum* [54], *M. velutinum* [44], *M. vulgare* [8,9,10,12,36,47,50]
64	NI-13	-	-	-	-	1587	0.1	-
65	Viridiflorol OST	Validation	1573.436	1597	0.1	-	-	*M. aschersonii* [34], *M. astracanicum* [38], *M. crassidens* [38], *M. incanum* [51], *M. parviflorum* [47], *M. peregrinum* [43], *M. vulgare* [10,12,34,47]
66	Hexadecane A	Train	1594.576	1602	0.1	-	-	*M. duabense* [40], *M. velutinum* [44]
67	Humulene epoxide II OST	Train	1626.959	1607	0.2	1607	0.2	*M. anisodon* [37], *M. incanum* [51], *M. thessalum* [54], *M. vulgare* [10]
68	Muurola-4,10(14)-dien-1-β-ol OST	Train	1605.330	-	-	1627	0.3	
69	NI-14	-	-	1628	0.1	-	-	-
70	4,4-dimethyl-Tetracyclo [6.3.2.0(2,5).0(1,8)]tridecan-9-ol O	Validation	1605.030	-	-	1631	0.2	
71	NI-15	-	-	1632	0.1	-	-	-
72	Caryophylla-4(12),8(13)-dien-5-α-ol OST	Train	1605.030	1636	0.1	1635	0.3	
73	epi-α-Muurolol (=tau-muurolol) OST	Test	1605.030	1642	0.2	1641	0.6	*M. astracanicum* [38], *M. deserti* [35], *M. incanum* [51], *M. parviflorum* [42], *M. peregrinum* [44], *M. velutinum* [44]
74	α-Muurolol (=Torreyol) OST	Train	1652.148	-	-	1645	0.1	
75	α-Cadinol OST	Train	1682.934	1654	0.3	1654	0.9	*M. crassidens* [38], *M. deserti* [35,52], *M. incanum* [50,51], *M. parviflorum* [42], *M. persicum* [45], *M. vulgare* [12,50]
76	NI-16	-	-	1658	0.2	1656	0.2	-
77	NI-17	-	-	1662	0.1	1662	0.1	-
78	E-Calamenen-10-ol OST	Train	1608.844	-	-	1669	0.1	
79	NI-18	-	-	1668	0.2	-	-	-
80	NI-19	-	-	-	-	1670	0.2	-
81	8-Heptadecene O	Train	1607.164	-	-	1673	0.2	
82	1-Tetradecanol O	Train	1702.771	1675	0.1	-	-	
83	Germacra-4(15),5,10(14)-trien-1-α-ol OST	Train	1700.003	1682	0.1	1685	0.2	
84	Heptadecane A	Validation	1726.886	1696	0.3	1696	0.2	*M. anisodon* [37], *M. parviflorum* [42,47], *M. vulgare* [10,47]
85	Pentadecanal O	Validation	1581.928	1710	0.1	1711	0.1	*M. anisodon* [37]
86	Mint sulfide ST	Train	1778.777	1733	0.1	1736	0.1	
87	NI-20	-	-	1734	0.1	-	-	-
88	NI-21	-	-	1742	0.1	-	-	-
89	NI-22	-	-	1743	0.4	1744	0.1	-
90	E-3-Octadecene O	Train	1722.391	-	-	1777	0.1	
91	n-Pentadecanol O	Train	1787.022	1778	0.1	-	-	*M. parviflorum* [42]
92	NI-23	-	-	-	-	1782	0.1	-
93	Octadecane A	Validation	1950.093	1796	0.1	-	-	*M. parviflorum* [47], *M. peregrinum* [43], *M. vulgare* [47]
94	NI-24	-	-	1819	0.1	-	-	-
95	6,10,14-trimethyl-2-Pentadecanone O	Train	1915.818	1844	4.8	1842	0.5	*M. peregrinum* [44], *M. velutinum* [44], *M. vulgare* [10]
96	NI-25	-	-	1849	0.1	-	-	-
97	NI-26	-	-	1853	0.2	-	-	-
98	NI-27	-	-	1888	0.1	-	-	-
99	NI-28	-	-	1891	0.8	1891	0.1	-
100	Nonadecane A	Test	1869.346	1897	0.2	1897	0.2	*M. duabense* [40], *M. parviflorum* [47], *M. peregrinum* [43], *M. vulgare* [10,15,47]
101	NI-29	-	-	1904	0.1	1906	Trace	-
102	5E,9E-Farnesyl acetone OST	Train	1956.289	1916	0.3	1915	Trace	*M. thessalum* [54], *M. vulgare* [15]
103	NI-30	-	-	1918	Trace	1917	Trace	-
104	NI-31	-	-	1924	0.1	-	-	-
105	NI-32	-	-	-	-	1926	Trace	-
106	NI-33	-	-	1925	0.1	-	-	-
107	NI-34	-	-	1929	0.1	-	-	-
108	NI			1938	0.1	1940	Trace	
109	Hexadecanoic acid O	Validation	1995.491	1960	3.9	-	-	*M. parviflorum* [42], *M. peregrinum* [36], *M. vulgare* [36,47]
110	NI-35	-	-	1973	0.1	1974	Trace	-
111	Eicosane A	Train	2034.560	1997	0.2	1994	0.1	*M. parviflorum* [48]
112	NI-36	-	-	2001	0.1	-	-	-
113	E,E-Geranyl linalool OD	Train	2028.645	2027	1.6	-	-	*M. aschersonii* [34], *M. parviflorum* [42], *M. vulgare* [12,34]
114	3,7,11,15-tetramethyl-(E,E)-1,6,10,14-Hexadecatetraen-3-ol OD			-	-	2028	0.9	
115	Manool OD	Train	2064.196	2057	0.3	-	-	
116	NI-37	-	-	2061	0.1	-	-	-
117	NI-38	-	-	2067	0.1	-	-	-
118	NI-39	-	-	2084	0.1	-	-	-
119	NI-40	-	-	2096	0.1	-	-	-
120	Heneicosane A	Train	2120.284	2101	1.6	2100	1.3	*M. parviflorum* [42,47], *M. peregrinum* [43], *M. propinquum* [41], *M. vulgare* [10]
121	NI-41	-	-	2108	0.3	2105	0.2	-
122	NI-42	-	-	2112	0.2	2110	0.3	-
123	Phytol OD	Test	2124.818	2116	1.4	2113	0.4	*M. anisodon* [37], *M. incanum* [51], *M. parviflorum* [41,42], *M. peregrinum* [36], *M. propinquum* [41], *M. vulgare* [10,15]
124	NI-43	-	-	2131	0.2	-	-	-
125	NI-44	-	-	2143	0.2	2143	0.1	-
126	NI-45	-	-	2147	0.2	-	-	-
127	NI-46	-	-	2164	0.2	2160	0.2	-
128	NI-47	-	-	2167	0.1	2172	0.3	-
129	NI-48	-	-	2175	0.6	2176	0.4	-
130	NI-49	-	-	2181	0.9	2179	0.2	-
131	NI-50	-	-	2183	0.4	-	-	-
132	NI-51	-	-	2198	2.4	2195	2.4	-
133	Docosane A	Validation	2194.421	2205	0.9	2198	0.6	*M. crassidens* [39], *M. parviflorum* [47]
134	NI-52	-	-	-	-	2201	0.1	-
135	NI-53	-	-	-	-	2209	0.3	-
136	NI-54	-	-	2215	0.3	-	-	-
137	NI-55	-	-	2225	0.3	2221	0.1	-
138	NI-56	-	-	2246	0.3	-	-	-
139	NI-57	-	-	2258	0.2	2253	0.3	-
140	NI-58	-	-	2270	0.1	2265	0.1	-
141	NI-59	-	-	2277	0.2	2274	0.2	-
142	NI-60	-	-	2293	3.8	2288	1.7	-
143	Tricontane A	Train	2381.642	2305	3.6	2302	2.6	
144	NI-61	-	-	2309	0.2	2305	0.2	-
145	NI-62	-	-	2344	0.2	2341	0.3	-
146	NI-63	-	-	2380	0.1	2377	0.1	-
147	NI-64	-	-	2383	0.1	2382	0.1	-
148	Tetracosane A	Train	2493.491	2401	0.3	2395	0.2	*M. deserti* [52], *M. parviflorum* [41,42,47], *M. propinquum* [41]
149	NI-65	-	-	2454	0.4	2447	0.2	-
150	NI-66	-	-	2488	0.2	2483	0.2	-
151	Pentacosane A	Test	2510.087	2503	0.8	2497	0.6	*M. anisodon* [37], *M. parviflorum* [42,47]
152	Heptacosane A	Train	2730.537	2702	0.6	2696	0.5	*M. anisodon* [37], *M. aschersonii* [34], *M. incanum* [51], *M. parviflorum* [42], *M. vulgare* [34]
153	NI-67	-	-	-	-	2766	0.1	-
154	Octacosane A			2801	0.1	2791	Trace	*M. crassidens* [39], *M. parviflorum* [42], *M. persicum* [45]
155	Squalene T	Train	2870.673	2835	0.1	2823	0.1	
156	NI-68	-	-	2868	0.1	2855	0.1	-
157	Nonacosane A	Test	2930.732	2905	0.7	2892	0.6	*M. anisodon* [37], *M. crassidens* [39], *M. persicum* [45]
158	Untriacontane A	Validation	3150.673	3105	0.4	3095	0.3	
159	NI-69	-	-	-	-	3212	0.1	-
160	Tritriacontane A	Train	3319.753	3300	0.1	3301	Trace	
	Oxygenated monoterpenes OMN				0.1		0.2	
	Sesquiterpene hydrocarbons ST				52.0		67.8	
	Oxygenated sesquiterpenes OST				5.8		6.2	
	Oxygenated diterpenes OD				3.3		1.3	
	Triterpene T				0.1		0.1	
	Aromatics AR				0.4		0.4	
	Alkanes A				11.7		7.4	
	Other O				9.9		3.4	
	NI				16.7		12.5	
	Total				100		99.3	

RI_pred._—BRT calculated retention index; RI^a^—retention index experimentally obtained on a HP-5MS column; Other—aliphatic hydrocarbons, aliphatic aldehydes and alcohols, aliphatic acids, their esters and aldehydes, aromatic esters with aliphatic acids, alkyl-aromatic alcohols, or aryl esters of aromatic acids; NI—not identified compound.

**Table 2:** The correlation coefficient matrix for the selected descriptors by GA.

	AATSC2p	MATS5v	GATS6v	SM1_Dzv	VR1_Dzs	VAdjMat
AATSC4e	0.031	−0.138	−0.135	0.030	0.205	0.224
AATSC2p		−0.265	−0.131	0.036	−0.231	−0.010
MATS5v			0.212	−0.008	0.066	0.109
GATS6v				0.072	0.131	0.214
SM1 Dzv					0.058	0.084
VR1 Dzs						2.339

**Table 3:** The “goodness of fit” tests for the developed BRT model.

Boosted Tree Model	*χ* ^2^	*RMSE*	*MBE*	*MPE*	*r* ^2^
2019	4455.272	66.160	−13.063	3.285	0.956
2020	3975.751	62.581	−7.698	3.241	0.964

*χ*^2^—reduced chi-square, *MBE*—mean bias error, *RMSE*—root mean square error, *MPE*—mean percentage error.

## Data Availability

Not applicable.
